# Measurement of maternal mortality, United Republic of Tanzania

**DOI:** 10.2471/BLT.25.295623

**Published:** 2026-07-20

**Authors:** Ahmad Mohamed Makuwani, Sophia Kagoye, Honorati Masanja, Habib Rutakyamirwa Ismail, Rose Mpembeni, Josephine Shabani, Secilia Kapalata Ng’weshemi, Golden Masika, Leonard Katalambula, Ties Boerma, Claudia Hanson

**Affiliations:** a Department of Reproductive, Maternal and Child Health, Ministry of Health, United Republic of Tanzania.; b Institute for Global Public Health, University of Manitoba, Winnipeg, Canada.; c Ifakara Health Institute, Ifakara, United Republic of Tanzania.; d School of Public Health and Social Science, Muhimbili University of Health and Allied Science, Dar es Salaam, United Republic of Tanzania.; e School of Medicine and Dentistry, University of Dodoma, Dodoma, United Republic of Tanzania.; f School of Nursing and Public Health, University of Dodoma, Dodoma, United Republic of Tanzania.; g Department of Global Public Health, Karolinska Institutet, Widerströmska Huset, Tomtebodavägen 18a, 17165 Stockholm - Solna, Sweden.

## Abstract

Maternal mortality, defined as the death of a woman during pregnancy or shortly after childbirth, is a key target of the sustainable development goals. Yet, in many low- and middle-income countries, accurate measurement of maternal mortality remains a major challenge. Reliable death registration systems are often incomplete or absent, making it difficult to calculate the maternal mortality ratio. However, opportunities exist to improve measurement. In about two thirds of low- and middle-income countries, more than 80% of births now occur in health facilities. This shift means that health facilities can serve as a valuable source of data not only for local quality improvement but also for informing population mortality estimates. However, this requires high-quality data. To improve the quality of facility-based data, we propose four simple quality metrics: (i) plausibility of reported national and subnational levels and patterns; (ii) the ratio of reported stillbirths to maternal deaths; (iii) the distribution of causes of death; and (iv) the share of deaths among women aged 35 years and older. Using data from the United Republic of Tanzania as an example, we show how these metrics can be applied to assess the completeness and internal consistency of routine health data systems. Our example aims to encourage quality assessment of facility-based data in other countries, alongside: (i) individual death reporting using standardized classification of causes of death; (ii) centralized data quality assessment based on the proposed metrics; and (iii) regular national on-site assessment of the completeness and accuracy of maternal and perinatal death reporting.

## Résumé

La mortalité maternelle, définie comme le décès d’une femme en cours de grossesse ou peu après l’accouchement, constitue un point clé des Objectifs de développement durable. Pourtant, dans de nombreux pays à revenu faible ou intermédiaire, la mesure précise de cette mortalité reste un défi majeur. Les systèmes fiables d’enregistrement des décès sont souvent incomplets, voire inexistants, ce qui complique le calcul du taux de mortalité maternelle. Il existe toutefois des possibilités d’améliorer cette mesure. Dans environ deux tiers des pays à revenu faible ou intermédiaire, plus de 80% des naissances ont désormais lieu dans des établissements de soins de santé. Cette évolution implique que ces établissements peuvent constituer une source précieuse de données, non seulement pour l’amélioration de la qualité à l’échelle locale, mais aussi pour étayer les estimations de la mortalité de la population. Cette approche nécessite toutefois des données de haute qualité. Afin d’améliorer la qualité des données issues des établissements de soins de santé, nous proposons quatre indicateurs simples de la qualité: (i) la plausibilité des niveaux et des tendances signalés aux échelles nationale et infranationale; (ii) le rapport entre les mortinaissances signalées et les décès maternels; (iii) la répartition des causes de décès; et (iv) la part de décès chez les femmes âgées d’au moins 35 ans. En prenant comme exemple les données de la République-Unie de Tanzanie, nous mettons en évidence la manière d’utiliser ces indicateurs pour évaluer l’exhaustivité et la cohérence interne des systèmes de données de soins de santé de routine. Notre exemple vise à encourager l’évaluation de la qualité des données issues des établissements de soins de santé dans d’autres pays, parallèlement à: (i) la déclaration individuelle des décès à l’aide d’une classification standardisée des causes de décès; (ii) une évaluation centralisée de la qualité des données fondée sur les indicateurs proposés; et (iii) une évaluation nationale régulière, sur place, de l’exhaustivité et de l’exactitude des déclarations de décès maternels et périnataux.

## Resumen

La mortalidad materna, definida como la muerte de una mujer durante el embarazo o poco después del parto, constituye una meta fundamental de los Objetivos de Desarrollo Sostenible. Sin embargo, en muchos países de ingresos bajos y medios, la medición precisa de la mortalidad materna sigue representando un importante desafío. Los sistemas fiables de registro de defunciones suelen ser incompletos o inexistentes, lo que dificulta el cálculo de la razón de mortalidad materna. No obstante, existen oportunidades para mejorar su medición. En aproximadamente dos tercios de los países de ingresos bajos y medios, más del 80% de los partos tienen lugar actualmente en establecimientos de salud. Este cambio implica que dichos establecimientos pueden constituir una valiosa fuente de datos, no solo para la mejora de la calidad a nivel local, sino también para fundamentar las estimaciones de la mortalidad poblacional. Sin embargo, ello requiere datos de alta calidad. Para mejorar la calidad de los datos procedentes de los establecimientos de salud, se proponen cuatro métricas sencillas de calidad: (i) la plausibilidad de los niveles y patrones nacionales y subnacionales notificados; (ii) la razón entre las muertes fetales notificadas y las muertes maternas (iii) la distribución de las causas de muerte; y (iv) la proporción de defunciones entre mujeres de 35 años o más. Utilizando datos de la República Unida de Tanzanía como ejemplo, se muestra cómo estas métricas pueden aplicarse para evaluar la exhaustividad y la consistencia interna de los sistemas rutinarios de información sanitaria. El objetivo de este ejemplo es fomentar la evaluación de la calidad de los datos procedentes de los establecimientos de salud en otros países, junto con: (i) la notificación individual de las defunciones mediante una clasificación estandarizada de las causas de muerte; (ii) la evaluación centralizada de la calidad de los datos basada en las métricas propuestas; y (iii) la evaluación nacional periódica sobre el terreno de la exhaustividad y la exactitud de la notificación de las muertes maternas y perinatales.

## ملخص

يتم تعريف وفيات الأمهات بأنها وفاة المرأة أثناء الحمل، أو بعد الولادة بفترة وجيزة، وهي هدف رئيسي من أهداف التنمية المستدامة. ومع ذلك، لا يزال القياس الدقيق لوفيات الأمهات يُمثل تحديًا كبيرًا في العديد من الدول ذات الدخل المنخفض والمتوسط. وغالبًا ما تكون أنظمة تسجيل الوفيات الموثوقة غير مكتملة أو غير قائمة، مما يجعل من الصعب حساب نسبة وفيات الأمهات. إلا أنه هناك فرص لتحسين القياس. ففي حوالي الثلثين من الدول ذات الدخل المنخفض والمتوسط، تتم أكثر من %80 من الولادات الآن في المرافق الصحية. ويعني هذا التحول أن المرافق الصحية يمكن أن تمثل مصدرًا مهمًا للبيانات، ليس فقط لتحسين الجودة على المستوى المحلي، بل أيضًا للتعرف على تقديرات وفيات السكان. إلا أن هذا يتطلب بيانات عالية الجودة. ولتحسين جودة البيانات المستمدة من المرافق الصحية، فإننا نقترح أربعة معايير بسيطة للجودة: (1) مدى معقولية المستويات والأنماط الوطنية ودون الوطنية المُبلَّغ عنها؛ و(2) نسبة حالات وفيات الرضّع المُبلَّغ عنها إلى وفيات الأمهات؛ و(3) توزيع أسباب الوفاة؛ و(4) نسبة الوفيات بين النساء اللاتي تبلغ أعمارهن 35 عامًا فأكثر. باستخدام بيانات من جمهورية تنزانيا المتحدة كمثال، فإننا نوضح كيفية تطبيق هذه المعايير لتقييم مدى اكتمال واتساق أنظمة البيانات الصحية الروتينية. يهدف مثالنا إلى تشجيع تقييم جودة البيانات المستمدة من المرافق الصحية في دول أخرى، إلى جانب: (1) الإبلاغ عن حالات الوفاة الفردية باستخدام تصنيف موحد لأسباب الوفاة؛ و(2) التقييم المركزي لجودة البيانات بناءً على المعايير المقترحة؛ و(3) التقييم الوطني الدوري في الموقع لمدى اكتمال ودقة الإبلاغ عن وفيات الأمهات والمواليد.

## 摘要

孕产妇死亡指女性在妊娠期或分娩后短期内发生的死亡，是可持续发展目标的核心考核指标。然而，在许多中低收入国家，准确测算孕产妇死亡情况仍然是一项巨大的挑战。可靠的死亡登记系统往往不完善或根本不存在，致使孕产妇死亡比难以测算。但目前仍有完善测算工作的机会。在约三分之二中低收入国家，目前超过 80% 的分娩均在医疗机构内完成。这一转变意味着医疗机构可作为重要数据来源，既能用于当地医疗质量改进，也能为人群死亡情况的估算提供依据。然而，这需要优质的数据。为了提高机构数据的质量，我们提出了四个简单的质量指标：(i) 全国及国家以下各级行政区域上报数据水平与变化模式的合理性；(ii) 上报死产数与孕产妇死亡数的比值；(iii) 死亡原因构成分布；以及 (iv) 35 岁及以上孕产妇死亡占比。我们以坦桑尼亚联合共和国的数据为例，阐释如何运用上述指标评估常规卫生数据系统的数据完整性与内部一致性。本文案例旨在推动其他国家开展医疗机构数据质量评估，除此之外，还应配套落实以下措施：(i) 采用标准化死亡原因分类开展个案死亡上报；(ii) 基于上述指标开展集中式数据质量评估；以及 (iii) 定期在全国范围内实地核查孕产妇及围产儿死亡上报数据的完整性与准确性。

## Резюме

Снижение материнской смертности, определяемой как смерть женщины во время беременности или вскоре после родов, является ключевым показателем целей в области устойчивого развития. Тем не менее во многих странах с низким и средним уровнем дохода точная оценка материнской смертности остается серьезной проблемой. Надежные системы регистрации случаев смерти часто отсутствуют или регистрируются не все случаи смертности, что затрудняет расчет коэффициента материнской смертности. Однако существуют возможности для улучшения оценки. В большинстве (примерно 2/3) стран с низким и средним уровнем дохода более 80% родов сейчас происходят в медицинских учреждениях. Благодаря этой тенденции медицинские учреждения могут стать ценным источником данных не только для повышения качества медицинской помощи на местном уровне, но и для определения оценок смертности населения. Однако для этого требуются высококачественные данные. Чтобы повысить качество данных на базе медицинских учреждений, авторы предлагают использовать четыре простых показателя качества: (i) достоверность сообщаемых национальных и субнациональных уровней и закономерностей; (ii) соотношение числа зарегистрированных случаев мертворождения к числу материнских смертей; (iii) распределение причин смерти; (iv) доля смертей среди женщин в возрасте 35 лет и старше. На примере данных по Объединенной Республике Танзания авторы показывают, как эти показатели можно применять для оценки полноты и внутренней согласованности стандартных систем обработки данных в сфере здравоохранения. Данный пример направлен на то, чтобы стимулировать оценку качества данных на базе медицинских учреждений в других странах, а также предоставить следующие данные и возможности: (i) индивидуальную отчетность для смертности с использованием стандартизированной классификации причин смерти; (ii) централизованную оценку качества данных на основе предложенных показателей; (iii) регулярную национальную выездную оценку полноты и точности отчетности о материнской и перинатальной смертности.

## Introduction

Measuring maternal mortality, defined as death in pregnancy, childbirth and the six weeks thereafter, is a key indicator for reviewing progress towards the sustainable development goals, as well as for national health sector reviews.^1^ At the global level, the United Nations Maternal Mortality Estimation Inter-Agency Group provides estimates in 2–3 years intervals.^2^^,^^3^ Uncertainty intervals for the estimates are often large, due to the limited underlying data. In addition, the Global Burden of Disease (GBD) publishes global, regional and national estimates, which often differ considerably from the Maternal Mortality Estimation Inter-Agency Group estimates.^4^ These discrepancies raise concerns about the accuracy of these estimates.^5^ Data sources for these estimates include death registration as part of civil registration and vital statistics systems, reproductive age mortality studies, population census and household surveys.^2^

While civil registration and vital statistics systems are considered the best source for mortality estimates, reliable systems are lacking in most low- and lower-middle income countries. As a result, surveys and sometimes censuses are the main sources for these estimates.^2^^,^^3^^,^^6^ Large household surveys, such as the Demographic and Health Surveys (DHS) and Multiple Indicator Cluster Surveys, typically rely on women reporting on the survival of their siblings to estimate adult and maternal mortality.^2^^,^^7^^,^^8^ However, this approach has important limitations. Maternal deaths are relatively rare events, leading to small sample sizes and wide uncertainty intervals. In addition, estimates are based on long reference periods, often seven years before the survey, which makes it difficult to track recent changes. Declining fertility and mortality rates, along with increasing migration, further challenge the accuracy of the sibling-based method.^9^^,^^10^ Population censuses may provide additional data points, providing information about household deaths in the last 12 or 24 months, although the quality of these estimates is highly variable.^11^

These limitations, and the inability of survey data to provide subnational estimates, have not only called for improvements in civil registration and vital statistics in low- and middle-income countries, but also increased interest in using health facility data, as the majority of births take place in health facilities. Using health facility data would be of major value. Evidence already exists that combined use of population and health facility data can improve estimates of population indicators for immunization coverage,^12^ family planning^13^^,^^14^ and prevalence of human immunodeficiency virus.^15^^,^^16^

Incomplete reporting of maternal deaths by health facilities is, however, a major concern. We therefore argue that a methodological approach and roadmap to improve facility death reporting should be developed to improve the quality of facility-based maternal mortality estimates, with the goal of enabling their integration into global estimates of population mortality. Here we propose four data quality metrics to review facility-based maternal mortality data, assessing the plausibility of: (i) levels and ranking of institutional maternal mortality ratios; (ii) the ratio of stillbirths to maternal deaths; (iii) cause-of-death distribution; and (iv) age distribution (Box 1). Our work, including the case study presented, was developed based on insights generated from two workshops conducted in 2024, bringing together stakeholders involved in maternal mortality measurements in the United Republic of Tanzania. The work was organized as part of the Countdown to 2030 initiative and is stimulating similar work in other settings.^17^

Box 1 **Proposed quality metrics to assess completeness of health facility data on maternal mortality**

***I.* *Plausibility of levels and ranking of institutional maternal mortality ratio***

No district or other administrative entity with a hospital should have an institutional maternal mortality ratio below 25 deaths per 100 000 live births; subnational ranking should align with facility birth coverage, poverty levels and other related development indicators.

***II. Plausibility of the stillbirth-to-maternal death ratio***

Fewer than 10% of subnational units shall have a ratio of stillbirths to maternal deaths below 6 or above 15.

***III. Plausibility of cause-of-death distribution***

The proportion of deaths due to haemorrhage, hypertensive disorders and obstructed labour should be < 60%, with reporting of abortion and pregnancy-related infections of > 5%.

***IV. Plausibility of age distribution***

The proportion of deaths in women  35 years and older should be at least 1.5 times the share of births in the population.

## Facility maternal death data

Facility maternal death data are typically generated through multiple data systems. Data sources include: (i) the District Health Information System 2 (DHIS2), which operates in over 80 low- and middle-income countries;^18^ (ii) the Maternal and Perinatal Death Surveillance and Response system, which captures maternal and perinatal deaths for surveillance and audit purposes;^19^ and (iii) research studies based on data from individual or multiple health facilities. We refer to maternal mortality derived from health facility data as the institutional maternal mortality ratio, defined as the number of maternal deaths per 100 000 live births occurring within the same facilities and time period.

## Quality metrics

Box 1 summarizes the operational thresholds for our four proposed quality metrics. Below we outline their rationale and interpretation. Further details are available in the online repository.^20^

### Subnational mortality ratio

We divided this metric into two submetrics: metric Ia assesses the plausibility of absolute levels, whereas metric Ib examines whether relative differences between subnational units are consistent with expected patterns. Based on empirical estimates,^2^ for metric Ia we deemed institutional maternal mortality ratios at a subnational level of less than 25 deaths per 100 000 live births, which is twice the level of high-income countries, to be implausible for low- and middle-income countries. However, this assumption applies only to administrative units with a hospital because of referral practices. For example, in United Republic of Tanzania less than 5% of districts do not have a hospital, implying that the remaining 95% of districts should have levels higher than 25 deaths per 100 000 live births.

For metric Ib, we expect subnational units with higher fertility, lower institutional delivery coverage and less-developed health services to have a higher institutional ratio than units with a better health infrastructure, based on available evidence from ecological studies.^21^^,^^22^

### Stillbirths to maternal deaths

Given the considerable overlap in the causes and underlying pathways of stillbirths and maternal deaths, we considered a ratio of stillbirths to maternal deaths between 6 and 15 to be plausible.^17^^,^^23^ Because some subnational units lack a hospital and/or referral practices or have a small population, we assumed that not more than 10% of subnational units should fall outside this range. Widespread deviation beyond this proportion may indicate inconsistencies in reporting.

### Cause of death distribution

Based on systematic reviews of causes of maternal death, we assume that about half are attributable to haemorrhage, hypertensive disorders and obstructed labour or ruptured uterus.^4^^,^^24^ Higher proportions in institutional data suggest underreporting of deaths occurring outside the labour ward, such as deaths due to abortion and pregnancy-related infection, which each account for 7–8% of maternal deaths, as well as indirect causes (25%).^24^ Thus subnational units reporting distributions that are inconsistent with expected patterns are likely to underreport deaths occurring outside the labour ward, particularly in early pregnancy or the postpartum period.

### Age distribution

Pregnant women older than 35 years have a two- to threefold increase in risk of dying compared to younger women.^25^^,^^26^ We therefore propose that the proportion of maternal deaths among women older than 35 years should be at least 1.5 times the share of births in the population. A lower proportion may indicate underreporting of deaths among older women, which are often associated with indirect causes.

## Case study

To show how these metrics can be used, we apply these quality metrics to data from the United Republic of Tanzania. We chose this country as the government has a strong interest in improving maternal mortality measurements to better monitor their policies and strategies. The health ministry has established a policy and strategy framework^27^ and invested in scaling up of health centres providing caesarean section services.^28^

### Local setting

The most recent United Nations estimates suggest a maternal mortality ratio of 276 deaths per 100 000 live births (80% uncertainty interval, UI: 192–429) in 2023, which is a major reduction from 486 deaths per 100 000 live births in 2010.^2^ The 2023 GBD estimate is 142 deaths per 100 000 live births (95% UI: 91–212).^4^ The national weighted proportion of institutional births has increased rapidly from 65% in 2015 to 81% in 2022, ranging from 72% in northern United Republic of Tanzania to nearly 100% in the southern highlands and Dar-es-Salaam. The total fertility rate was 4.8 births per woman in the 3 years before the 2022 DHS, with wide subnational variations.^29^

### Available data

#### Population-based data

The available data sources include DHS surveys (2010; 2015–16; 2022),^29^^–^^31^ census data (2012 and 2022)^32^^,^^33^ and subnational sources. The DHS 2022 estimated a maternal mortality ratio of 104 deaths per 100 000 live births (95% CI: 59–149) for 2016–2022 based on 30 reported deaths.^29^ Women older than 35 years accounted for 14.2% of all maternal deaths.^29^ The 2022 census data provides a maternal mortality ratio of 177 deaths per 100 000 live births based on 5180 pregnancy-related deaths, which has been derived from the crude estimate of 147 deaths per 100 000 live births in the national report.^3^^,^^34^

Population maternal mortality ratio estimates from the Magu Health and Demographic Surveillance Site in north-west United Republic of Tanzania suggest 280 deaths per 100 000 live births (2015–2022).^35^ In addition, a reproductive age mortality study conducted in Dar-es-Salaam estimated a maternal mortality ratio of 239 per 100 000 live births based on 848 maternal deaths (2019–2020).^36^ Verbal autopsy as part of the routine of civil registration and vital statistics and DHIS2 has been piloted in selected regions but reliable information on maternal mortality is not yet available.^37^

**Fig. 1:**
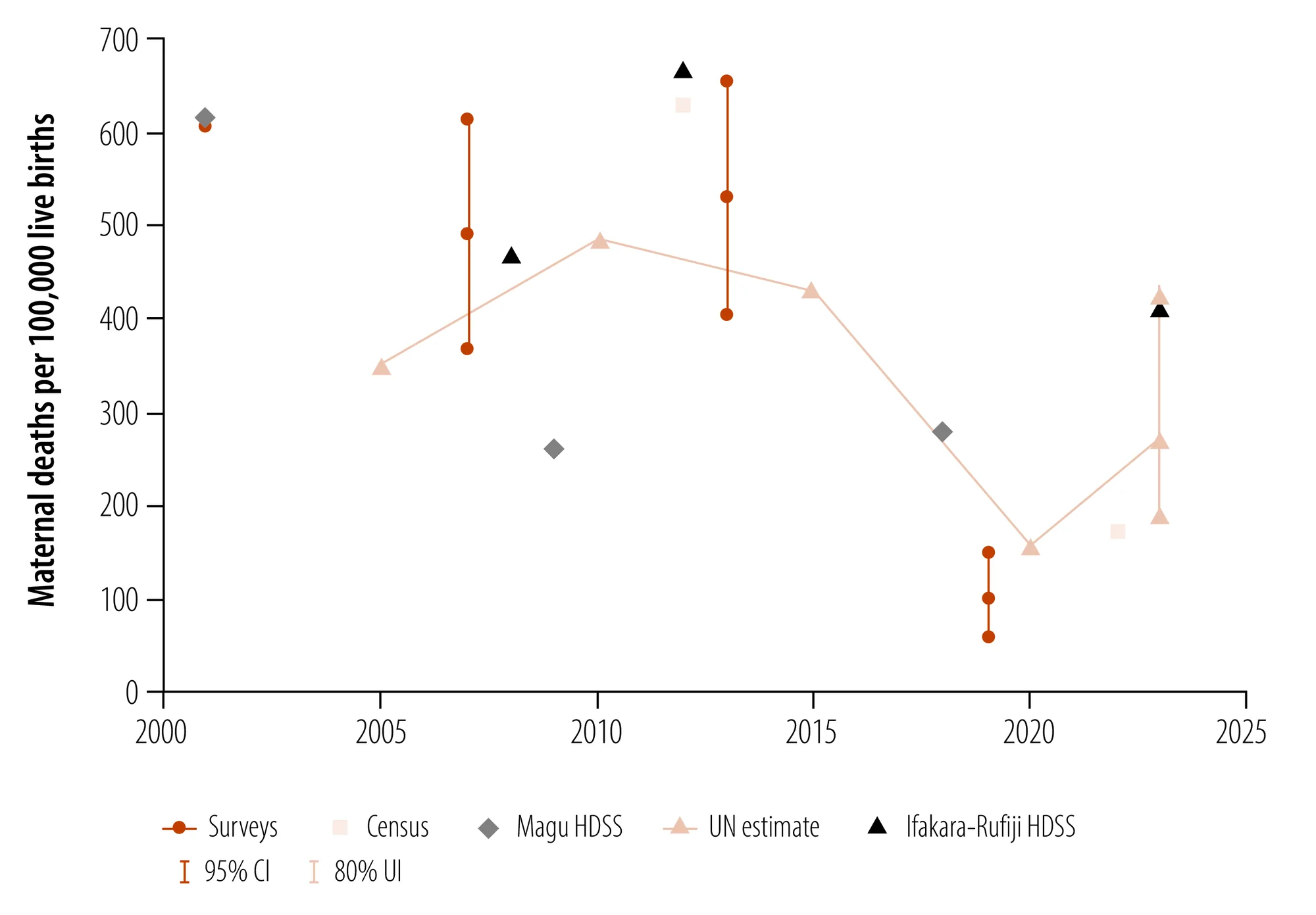
National and subnational population-based maternal mortality estimates, United Republic of Tanzania, 2002–2023

CI: confidence interval; UI: uncertainty interval; UN: United Nations.

As shown in Fig. 1, these many measurements create uncertainty in the population estimates and highlight the problem of underreporting on institutional deaths.

Data sources: We obtained the UN estimates and census data for 2012 and 2022 from WHO 2025.^3^ We obtained survey data from published DHS reports,^29^^–^^31^ Magu HDSS,^35^ and Ifakara-Rufiji Health & Demographic Surveillance System.^38^

#### Facility-based data

Available routine facility-based data sources on maternal deaths include the monthly district aggregated DHIS2 data^18^ and aggregated reports from the Maternal and Perinatal Death Surveillance and Response system at district and regional levels.^39^ In addition, we performed a systematic search in June 2025 using the keywords “(pregnancy OR birth OR childbirth OR mother* OR pregnan* OR maternal OR parturition OR ante-partum OR antepartum OR antenatal* OR intra-partum OR intrapartum OR peri-partum OR peripartum OR postpartum OR postpartum OR delivery) AND (death OR deceased OR mortality) AND (United Republic of Tanzania).” We searched for studies published since the year 2000 in PubMed® and Web of Science. We retrieved 32 studies reporting health facility data. We excluded population-based studies. Further information is available in the online repository.^20^

**Fig. 2:**
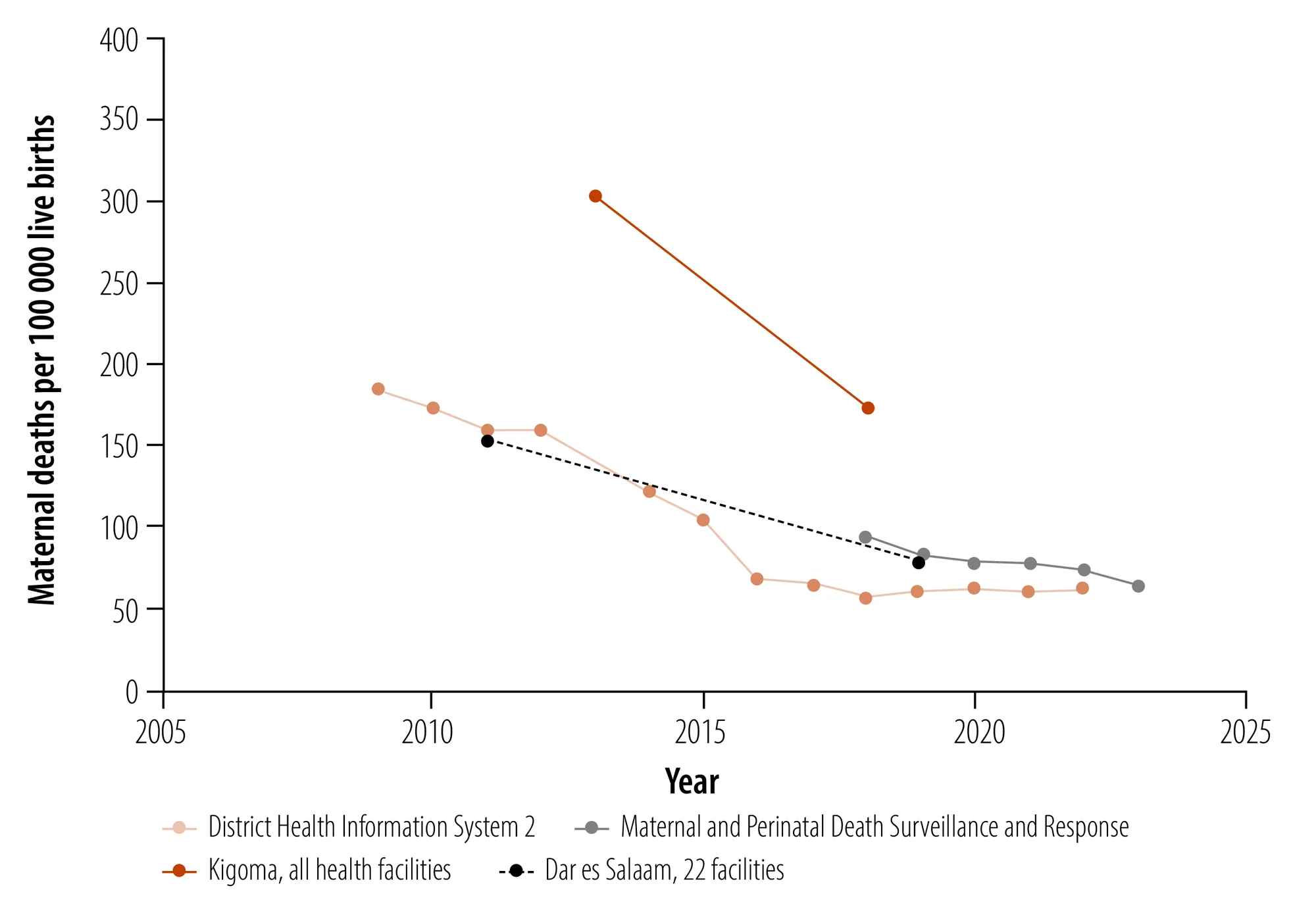
Health facility maternal mortality estimates, United Republic of Tanzania, 2009–2023

In the past 3 years, DHIS2 recorded 3582 deaths, while in the past 6 years the Maternal and Perinatal Death Surveillance and Response system recorded 8998 deaths. The institutional maternal mortality ratio based on data from DHIS2 and the surveillance and response system is 65 and 78 deaths per 100 000 live births, respectively (Table 1 and Fig. 2). The institutional maternal mortality ratio of published studies range from 45 in upgraded health centres providing caesarean section services^42^ to 1541 deaths per 100 000 live births in the national referral hospital in 2011 (Table 1).^43^

**Table 1:** Comparison of quality of maternal mortality facility data, United Republic of Tanzania, 2000–2023

Metric, plausible value for United Republic of Tanzania	DHIS2 (2020–2022)	Maternal and Perinatal Death Surveillance and Response system (2018–2023)	Research studies
**Ia. Plausibility of institutional maternal mortality ratio levels**			
< 5% of districts are expected to have institutional maternal mortality ratio below 25 as they have no hospital	37 (20.1%) of 184 districts report a ratio below 25	16 (8.7%) of 184 districts report a ratio below 25	NA
**Ib. Plausibility of the subnational institutional maternal mortality ratio rankings**			
Higher institutional birth coverage and better infrastructure should be associated with lower institutional maternal mortality ratio and vice versa	Limited plausibility in the data	Limited plausibility, regions with high institutional birth rate have higher institutional maternal mortality ratio	NA
**II. Plausibility of the stillbirth-to-maternal death ratio**			
< 10% of districts or other subnational units are expected to lie outside the range of 6 to 15	26 (14%) of 184 district report a ratio below 6 and 68 (37%) districts report above 15	22 (85%) of 26 regions report a ratio below 6, probably due to stillbirth underreporting and none above 15	NA
**III. Plausibility of cause-of-death distribution**			
< 60% of deaths are due to haemorrhage, hypertensive disorders, and obstructed labour causes > 5% of deaths should be due to abortion > 5% of deaths should be due to infection	NA^a^	61.5% of maternal deaths are due to the three main causes. 1.6% of deaths were attributed to abortion 6.8% deaths due to puerperal sepsis	15 (46.9%) of 32 studies report on cause of death, 11 (73.3%) of these had < 60% of deaths due to the three causes and report > 5% due to abortion or infection
**IV. Plausibility of age distribution**			
As 17% of births are in women aged 35 years or older, we expect 25.5% of deaths in this age group	NA^b^	19.3% of reported deaths were in women aged 35 years or older	9 (28.1%) of 32 studies reported results by age-group, five studies reported that > 25.5% of births are in women aged 35 years and older

NA: not applicable.

^a^ Cause of death data are not available from DHIS2 as only the aggregated number of maternal deaths per month are reported.

^b^ Age distribution of deaths are not available from DHIS2 as only the aggregated number of maternal deaths per month are reported

Note: definitions of the metrics are available in Box 1. The Maternal and Perinatal Death Surveillance and Response system only provided percentage for metrics III and IV. We obtained the proportion of births in women 35 years or older from the 2022 Demographic Health Survey.^29^

Data sources: data are from DHIS2, Maternal and Perinatal Death Surveillance and Response,^39^ Prasad et al., 2022,^40^ and Sequeira Dmello et al., 2021.^41^

##### Data quality

**Fig. 3:**
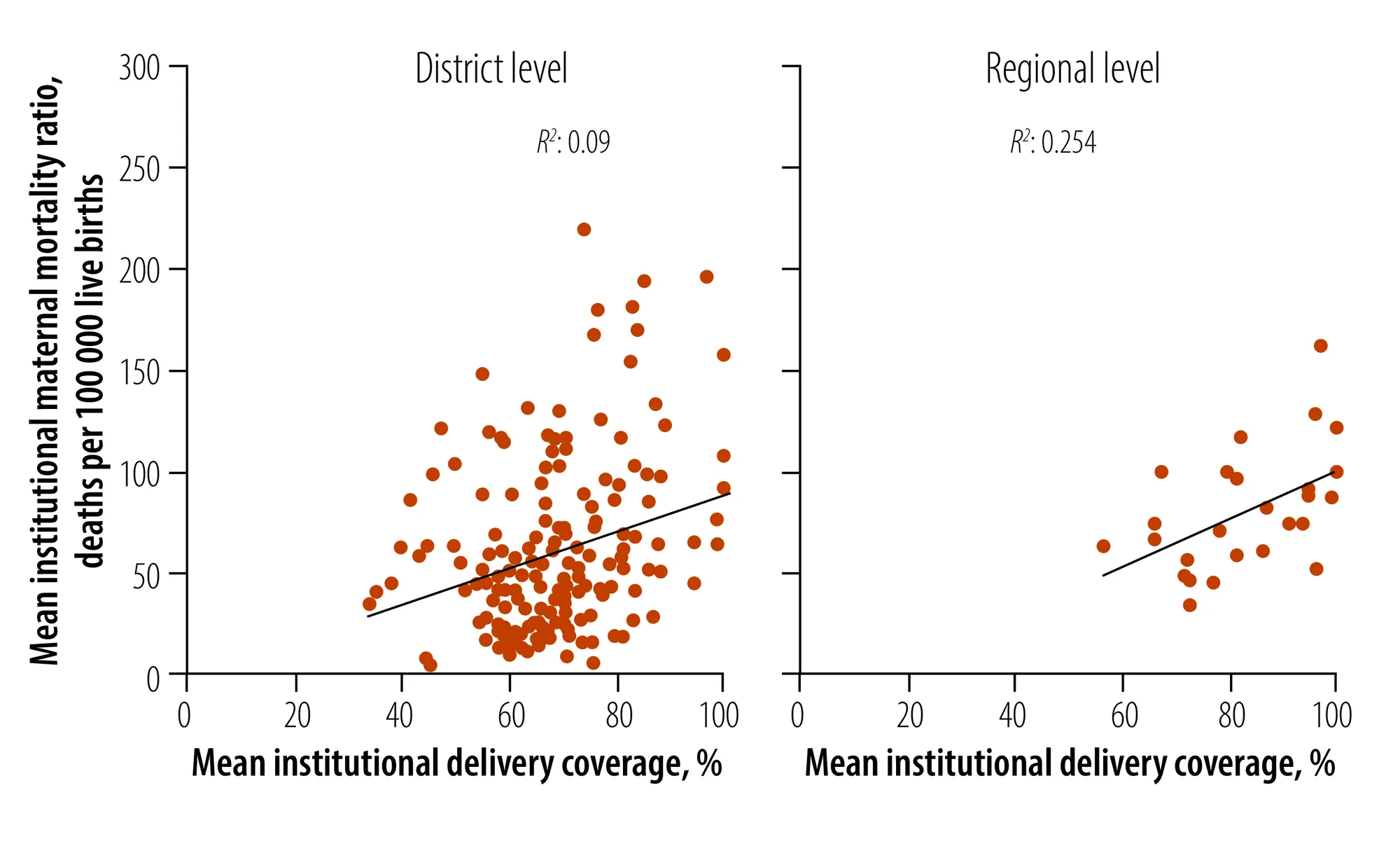
District and regional patterns of facility births versus maternal mortality ratio, United Republic of Tanzania, 2018–2023

Using our proposed quality metrics, we observe that for metric Ia, 20.1% (37/184) and 8.7% (16/184) of districts report implausibly low institutional maternal mortality ratios in DHIS2 and the Maternal and Perinatal Death Surveillance and Response system, respectively (Table 1). District and regional patterns contradict expected rankings based on access to facility births, which reflect socioeconomic development and health infrastructure, and therefore fail to meet the quality metric Ib: plausibility of subnational ranking. When analysing the relationship between institutional birth rates and institutional maternal mortality ratio using DHIS2 data, a higher institutional maternal mortality ratio was observed in places with higher institutional birth rates. A similar picture was observed for the Maternal and Perinatal Death Surveillance and Response system data, but fewer regions had very low institutional maternal mortality (Fig. 3; available at: https://www.who.int/publications/journals/bulletin/). 

Data sources: district data from DHIS2 (2020–2022) and regional data from the Maternal and Perinatal Death Surveillance and Response system (2018–2023).^39^

For metric II, DHIS2 data showed that 26 (14.1%) of 184 districts had a stillbirth to maternal death ratio below 6, and 68 (37.0%) above 15 (online repository).^20^ Using Maternal and Perinatal Death Surveillance and Response system data, 22 of 26 regions had a ratio below 6 indicating potentially major underreporting of stillbirth in this system (online repository).^20^

Data for assessing the plausibility of cause-of-death distribution (metric III) are only available for the Maternal and Perinatal Death Surveillance and Response system as DHIS2 does not record causes of death. The system attributed 61.5% of maternal deaths to the three causes of haemorrhage, hypertensive disorders and obstructed labour, indicating some underreporting of deaths outside the labour ward. Only 1.6% of deaths were attributed to abortion, 0.6% ectopic pregnancy, and 6.8% to puerperal sepsis. Roughly half (15/32) of the facility-based research studies provided information on cause of death and of those, 73.3% (11/15) reported < 60% of deaths due to the three causes and > 5% due to abortion and infection, suggesting a reasonable cause-of-death pattern. 

The quality metrics on age distribution (metric IV) indicated that 19.3% of deaths reported in the Maternal and Perinatal Death Surveillance and Response system were in women 35 years or older, thus slightly less than expected.^39^ Nine research studies reported on age at death, and five of these reported that > 25.5% of deaths were in women 35 years and older.^44^^–^^48^

### Comparison of estimates 

**Fig. 4:**
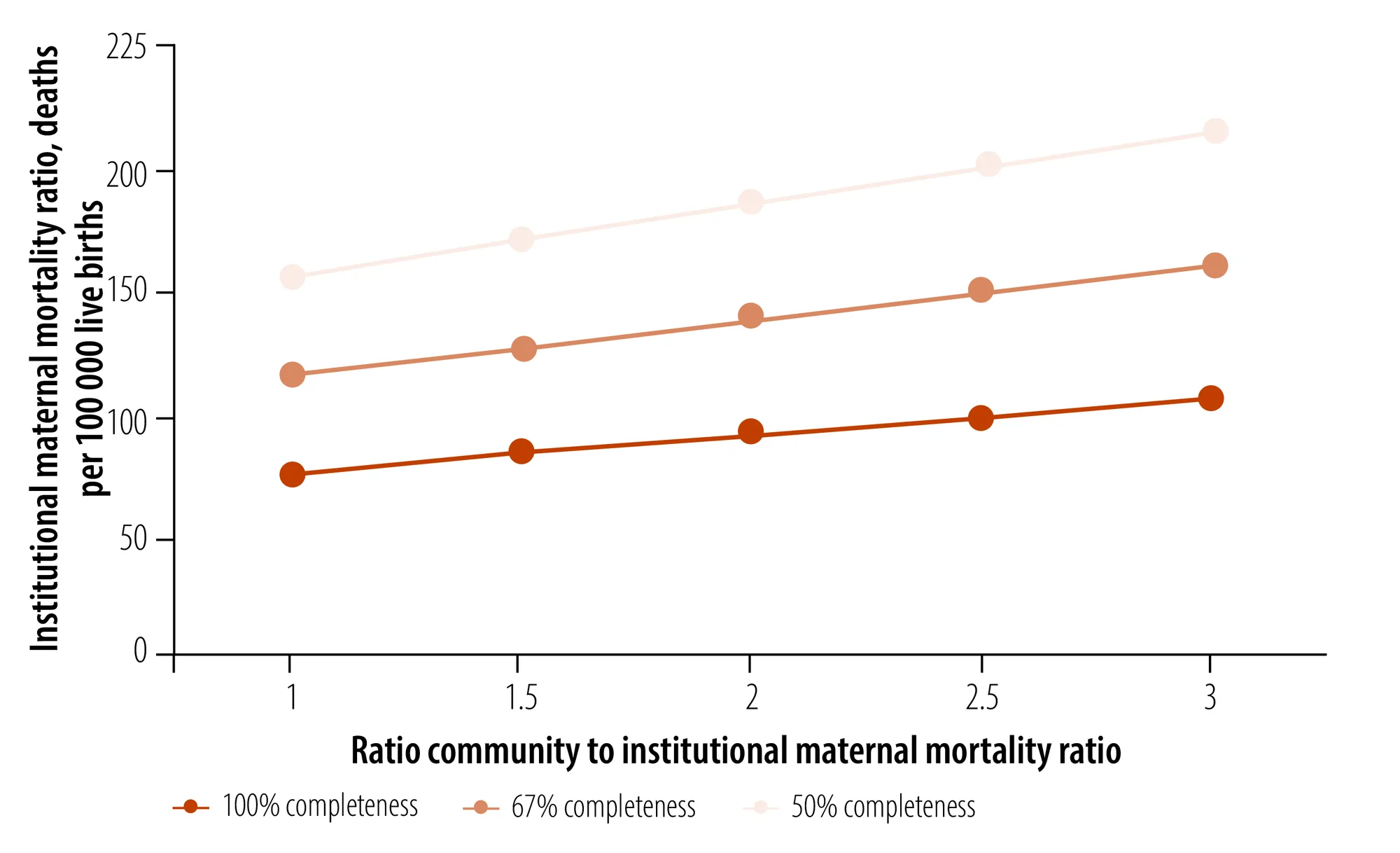
Institutional maternal mortality ratio based on different scenarios of community maternal mortality ratios and assumptions of reporting completeness, United Republic of Tanzania, 2018–2023

Note: based on the maternal mortality ratio from Maternal and Perinatal Death Surveillance and Response system,^39^ assuming with 100% completeness and a ratio of 1 for community to institutional maternal mortality ratio.

We compared the consistency of institutional maternal mortality ratio with population estimates, using scenarios with different assumptions about the completeness of the institutional maternal mortality ratio (100%, 66%, 50%) and the ratio of facility to community maternal mortality rate (ranging from 1 to 3), using published formulas (Fig. 4).^17^ We chose institutional maternal mortality ratio from the Maternal and Perinatal Death Surveillance and Response system as the data showed higher quality when assessed by our quality metrics (Table 1). We used the observed surveillance system institutional maternal mortality ratio of 78 deaths per 100 000 live births averaged over the past six years and the observed institutional birth rate of 81%. With 100% complete reporting by facilities and a community maternal mortality ratio double the institutional maternal mortality ratio, the population maternal mortality ratio would be 93. Assuming 50% reporting completeness, as observed in some studies in high-income countries, the population maternal mortality ratio would be 186. This estimate is substantially lower than the UN Maternal Mortality Estimation Inter-Agency Group maternal mortality ratio estimate of 276 deaths per 100 000 live births for 2023 but somewhat close to the group’s estimate for 2020, 160 deaths per 100 000 live births, and the 2023 GBD estimate of 142 deaths per 100 000 live births (95%: 91–212).^4^ Although population estimates have large uncertainty and temporal fluctuations, the comparison suggests that there is substantial underreporting in the Maternal and Perinatal Death Surveillance and Response system that needs to be addressed and quantified. For DHIS2 monthly reporting, incomplete reporting is more severe than in the surveillance system.

## Discussion

Here we propose quality metrics to assess whether available facility data reporting of maternal deaths is suitable for calculating institutional maternal mortality ratio. By applying the metrics on available facility data from United Republic of Tanzania, we observe quality and completeness issues across all four indicators and available data sources. Thus, these metrics provide insights into the completeness and quality of the reported institutional mortality data and their potential use for estimating population maternal mortality. At this point, we do not aim to define a universal gold standard for expected maternal mortality patterns, as these are inherently context-specific and vary across settings. Instead, we propose indicative ranges or thresholds to assess the plausibility of reported values. The four metrics should be interpreted jointly, as no single metric alone can provide a complete assessment of data quality.

Assuming a completeness of 50–67% in the facility data provides a reasonable adjustment for underreporting as the resulting maternal mortality estimates are consistent with levels from other population-based estimates, except for the 2023 estimate from the United Nations Maternal Mortality Estimation Inter-Agency Group. Differences between estimates are expected because incomplete reporting of maternal deaths is a global issue, and even estimates from civil registration and vital statistics in high-income settings are adjusted.^3^ Furthermore, the models and limited data used for estimation create large uncertainty in the estimates. Improving maternal mortality estimations requires both increasing completeness and robust estimation of completeness in facility data. In particular, efforts are needed to capture maternal deaths occurring outside maternity wards as the level of community maternal mortality is uncertain. Adding a single question regarding the place of death in sibling survival histories in household surveys would greatly help improve the assumptions used to estimate community maternal mortality ratio. 

We believe that regular quality assessment of facility data could improve reporting of maternal deaths. To ensure high-quality data, we propose four actions that facilities and health authorities can take. The first action is to improve the reporting of maternal deaths in facilities by emphasizing reporting in a single reporting system, thus assuring that DHIS2 and the Maternal and Perinatal Death Surveillance and Response system are linked and integrated (online repository).^20^ As the use of multiple unlinked reporting streams increases the reporting burden and system inefficiency,^49^^–^^51^ linking the systems is a first step to reduce underreporting. 

The second action is to establish regular national data quality reviews using the proposed metrics to assess completeness and internal consistency (Box 1). These regular data quality reviews should guide the implementation of improvement measures and support adjustments for incomplete reporting.

The third action is to develop and implement a system that independently verifies the completeness and accuracy of facility-reported maternal deaths. If resources permit, this system should be based on an annual assessment of a sample of health facilities using a standardized protocol. This assessment includes verifying the maternity registers, listing of all deaths among women of childbearing age in relevant wards (including internal medicine, surgical and gynaecological wards) and assessing the reported causes of death to identify missed maternal deaths. The main outcome of these assessments should be an estimate of reporting completeness that can be used to derive institutional- and population-level maternal mortality.

The fourth action is to estimate ranges for the population maternal mortality ratio, using institutional maternal mortality ratio and population data. These estimates should incorporate different scenarios of completeness of institutional maternal death reporting and varying assumptions about the ratio of community to institutional maternal mortality, given delivery coverage. The proportion of institutional births is widely available from surveys and DHIS2-based estimates.^52^ The community-to-institutional maternal mortality ratio can be obtained from research studies, but would improve if the sibling survival histories in surveys and mortality module in censuses included a question on place of death. Several countries, such as Sierra Leone, United Republic of Tanzania and Zimbabwe, are piloting or have established systems for civil registration and vital statistics reporting in the community. Data from these systems may further inform the community-to-institutional maternal mortality ratio.^37^^,^^53^^,^^54^

In addition, researchers should validate the assumptions to adjust for incompleteness used to calculate the community maternal mortality ratio.

## Conclusion

With this article, we wish to trigger further discussion on the use of facility death data, which is in line with national aspirations. We see a major opportunity given that the institutional birth rates in most low- and lower-middle income countries are increasing and often reach levels above 80%. Therefore, the institutional maternal mortality ratio can become a major input into the estimation of maternal mortality ratio at national and subnational levels.^52^ In addition, facility data may smoothen estimates, preventing an improbable upward trend, as modelled for the United Republic of Tanzania for the latest UN report. Facility maternal mortality data may also become the main source of estimates if DHS surveys do not resume after the current funding crisis.^55^

Regular assessment of reporting quality could improve maternal death reporting and, if data quality improves, may support their inclusion in the Maternal Mortality Estimation Inter-Agency Group estimates. Inclusion of such data could be based on quality criteria, which may incentivize improvements in both civil registration and vital statistics and facility-based maternal death reporting. Empirical studies are needed to assess the completeness and quality of reporting within facilities and to provide estimates of community deaths to inform evidence-based adjustments.
